# Association between anti-nuclear antibody (ANA) positivity and autoimmune thyroid disease markers in a general examination population

**DOI:** 10.1016/j.bbrep.2025.102330

**Published:** 2025-10-23

**Authors:** Mengjiao Yuan, Jinjin Wang, Wanjun Yu, Jianmin Lin, Lei Yue, Qian Gong

**Affiliations:** a Department of Clinical Laboratory, Qingpu Branch of Zhongshan Hospital, Fudan University, Shanghai, China; b Institute of Clinical Science, Zhongshan Hospital, Fudan University, Shanghai, China

**Keywords:** Anti-nuclear antibodies, Hashimoto's thyroiditis, Autoimmune thyroid antibodies, Thyroid hormone

## Abstract

**Background:**

Anti-nuclear antibodies (ANA), key biomarkers in systemic autoimmune diseases (AD), have been reported at higher levels in patients with autoimmune thyroid diseases (AITD), including Hashimoto's thyroiditis (HT) and Graves' disease (GD). However, a systematic evaluation of the association between ANA positivity and AITD serological markers (TPOAb, TgAb, TSH, fT3, fT4) in the general population is lacking.

**Methods:**

This retrospective cohort study analyzed data from 7556 individuals undergoing routine health examinations at our hospital. ANA was detected by indirect immunofluorescence. Thyroid markers were measured via chemiluminescence immunoassay. Participants were stratified by predefined clinical cut-offs for each marker. Associations between marker levels and ANA positivity, pattern and titer were analyzed respectively.

**Results:**

ANA positivity rates increased significantly with higher levels of TPOAb (≤34 IU/ml: 6.03 %, 34 IU/ml-100 IU/ml: 13.03 %, ≥100 IU/ml: 16.50 %; P < 0.001) and TgAb levels (≤115 IU/ml: 5.84 %, 115IU/ml-500IU/ml: 18.10 %, ≥500IU/ml: 19.29 %; P < 0.001). Logistic regression confirmed that with the increase in TPOAb and TgAb levels, the positive risk of ANA increased by approximately 1.6-fold and 2.7-fold respectively. TSH ≥4.2 mIU/L showed marginally increased ANA positivity (OR = 1.14, 95 % CI = 0.47–2.75). No significant associations were found with fT3 or fT4. AC-4/5 (speckled, 37.64 %) and AC-1 (homogeneous, 27.95 %) were dominant ANA patterns; the AC-1 prevalence increased with higher TPOAb/TgAb levels.

**Conclusion:**

Elevated thyroid autoantibodies (TPOAb, TgAb) are strongly associated with increased ANA positivity risk, suggesting a link between thyroid-specific and systemic autoimmunity. Thyroid hormone levels showed minimal association. Patients with high TPOAb/TgAb, especially females, may benefit from ANA screening.

## Highlights

• ANA positivity is dose-dependently linked to high TPOAb/TgAb.
• ANA is associated with elevated TSH, suggesting hypothyroidism risk.
• Homogeneous ANA pattern predominates in high-thyroid-antibody cases.
• ANA links specifically to Hashimoto's, not Graves' disease, markers.

## 1 Introduction

Anti-nuclear antibodies (ANA) are a type of auto-antibodies that target various nuclear antigens. ANAs can directly target nuclear and cytoplasmic proteins, nucleic acids and their complexes, so it can be used as a clinical biomarker of autoimmunity [1]. These biomarkers are very common in systemic autoimmune diseases (AD), such as systemic lupus erythematosus (SLE), rheumatoid arthritis (RA) and Sjogren's syndrome (SS), etc. [2, 3]. Therefore, ANA plays an important role in the diagnosis and classification of autoimmune diseases. The above-mentioned systemic AD are also often coexisting with organ-specific AD, such as autoimmune thyroid diseases (AITD), mainly comprising Graves' disease (GD) and Hashimoto's thyroiditis (HT). It has been reported that the risk of new-onset SLE in patients with HT has significantly increased [4]. Moreover, some studies have pointed out that HT and SLE have similar genetic features and are related to major histocompatibility complexes [5]. In addition, the thyroid function of patients with rheumatoid arthritis is also prone to decline [6]. This clinical overlap implies a potential link between the magnitude of thyroid-specific autoimmunity and the propensity for systemic autoimmunity.

The diagnosis of HT is mainly determined through biochemical tests (positive circulating thyroid autoantibodies) and imaging examinations [7]. Ninety percent of HT patients have relatively high levels of thyroid autoantibodies such as anti-thyroid peroxidase antibody (TPOAb) and thyroglobulin antibody (TgAb) [8, 9]. GD is usually identified by reduced serum Thyroid Stimulating Hormone (TSH) and increased production of thyroid hormones, specifically triiodothyronine (T3) and thyroxine (T4) [10, 11].

Previous studies have reported that compared with healthy people, the ANA levels of patients with ATID are also significantly higher [12, 13]. Conversely, high levels of TPOAb have also been observed in ANA-positive patients, such as those with SLE [14]. However, the precise quantitative relationship between specific, clinically relevant levels of thyroid biomarkers—including autoantibodies (TPOAb, TgAb) and hormones (TSH, fT3, fT4)—and ANA positivity remains incompletely elucidated in the general population. There is a lack of large-scale and rigorous analysis to determine how the classification levels of these parameters (based on established reference ranges and clinical thresholds) are related to ANA status and associated risks.

To address these crucial knowledge gaps, we conducted a large-scale retrospective cohort study. Our main objective was to determine the prevalence and risk of ANA positivity in a large clinical population across strictly defined levels of TPOAb, TgAb, TSH, fT3 and fT4. To explore the potential correlation between the levels of thyroid markers and the positive rate of ANA. Characterize the distribution of specific ANA immunofluorescence patterns and titers among key thyroid markers (TPOAb, TgAb, and TSH) at different concentration levels. This study aims to provide detailed and statistically reliable characterization of the serological interaction between thyroid autoimmunity/dysfunction and systemic autoimmunity, offering valuable insights for researchers and clinicians in managing patients with autoimmune diseases.

## 2 Materials and methods

### 2.1 Study design

This study adopts a retrospective design. Data from the included patients were extracted, organized and analyzed (Fig. 1). We included cases from August 16, 2022 to January 2, 2025, in the study. Our study cohort comprised individuals with available data on combinations of TPOAb and ANA, TgAb and ANA, TSH and ANA, fT3 and ANA or fT4 and ANA levels (These indicators are part of the health examination package offered to the physical examination population in our hospital). The study was authorized by the Medical Ethics Committee and it was to comply with the provisions of the declaration of Helsinki (Qingyi 2025-08). The study did not impede the personal privacy of patients and business interests.

**Fig. 1:**
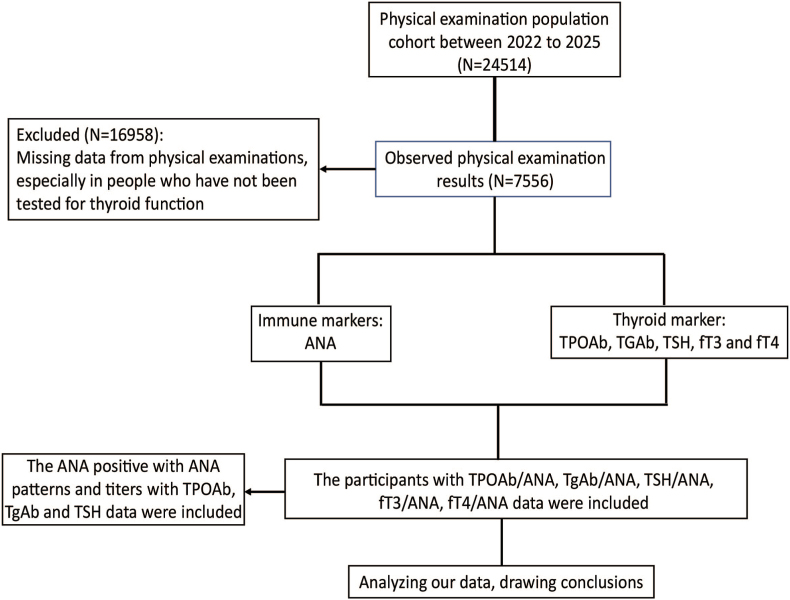
Study flowchart. ANA, antinuclear antibody; TPOAb, thyroid peroxidase antibodies; TgAb, thyroglobulin antibody; TSH, thyroid-stimulating hormone; fT3, free triiodothyronine; fT4, free thyroxine.

Inclusion criteria: Individuals who underwent both antinuclear antibody tests and thyroid marker tests during the physical examinations in our hospital from August 16, 2022 to January 2, 2025.

Exclusion criteria: Incomplete demographic or laboratory data, known diagnosis of tumors and systemic autoimmune diseases (e.g., SLE, RA) prior to enrollment, and use of immunosuppressive or immunomodulatory drugs.

### 2.2 ANA detection

Serum anti-nuclear antibodies (ANA) were detected using indirect immunofluorescence (IIF) on HEp-2 cells (Euroimmun kit). Undiluted serum samples were incubated on cell-coated slides (30 min, room temperature), washed with PBS, and then incubated with FITC-conjugated goat anti-human immunoglobulin (30 min, dark). Following a final PBS wash, slides were mounted. Positivity was defined as a titer ≥1:100; positive samples underwent titration (1:320, 1:1000). Slide images were acquired automatically (EUROPattern microscope) and evaluated both algorithmically (EPa software) and independently by two observers. Assessments included: 1. Negativity/Positivity; 2. Fluorescence Pattern: Classified as homogeneous, speckled, nucleolar, centromere, nuclear dot, nuclear membrane, or cytoplasmic, per international consensus nomenclature; 3. Titer: 1:100, 1:320, 1:1000, or >1:1000.

### 2.3 Thyroid hormone and antibody measurement

Serum TSH, fT3, fT4, TPOAb, and TgAb were quantified using electrochemiluminescence immunoassays (ECLIA) on a Cobas e801 analyzer (Roche Diagnostics). TSH was measured via sandwich ECLIA (LOD: 0.005 mIU/L; RI: 0.27–4.2 mIU/L). fT3, fT4, TPOAb, and TgAb were measured via competitive ECLIA (fT3 RI: 3.1–6.8 pmol/L; fT4 RI: 11.9–21.6 pmol/L; TPOAb RI: 0–34 IU/ml; TgAb RI: 0–115 IU/ml). Assay Precision (Intra- & Interassay CV): TSH, fT3, fT4: <8.33 %; TPOAb: <6.2 %; TgAb: <6.0 %. (Precision data derived from 10 QC cycles, 15–110 runs/cycle, spanning 4 representative months over 4 years).

### 2.4 Variable definitions

Our primary outcome **was** assessing ANA positivity and negativity rates across various TPOAb, TgAb, TSH, fT3 and fT4 levels. We subsequently calculated the risk of ANA positivity for different levels of the above indicators. In the primary analysis, TPOAb, TgAb, TSH, fT3 and fT4 levels were categorized based on the normal reference ranges provided by the hospital laboratory department and the target levels recommended by the guidelines. TPOAb levels 1, 2, and 3 were defined as ≤ 34, 34 < TPOAb <100, and TPOAb ≥100 IU/ml, respectively. TgAb levels 1, 2, and 3 were defined as ≤ 115, 115 < TgAb <500, and TgAb ≥500 IU/ml, respectively. TSH levels 1, 2, and 3 were defined as ≤ 0.27, 0.27 < TSH <4.2, and TSH ≥4.2 mIU/L, respectively. fT3 levels 1, 2, and 3 were defined as ≤ 3.1, 3.1 < fT3 < 6.8, and fT3 ≥ 6.8 pmol/L, respectively. fT4 levels 1, 2, and 3 were defined as ≤ 12, 12 < fT4 < 22, and fT4 ≥ 22 pmol/L, respectively.

### 2.5 Data analysis and statistical tests

Statistical analyses were performed using SPSS version 25.0 (IBM Corp.; Armonk, NY, USA). The non-normal distributed continuous variables were expressed as median with interquartile range (IQR) and compared with the Mann-Whitney *U* test. Categorical variables were expressed as numbers and percentages and were compared by the Chi-square test (or Fisher's exact test if required). Kendall's tau-b analysis was also performed for the inter-indicator correlation. Logistic regression analysis was employed to determine the risk factor for ANA positivity. P < 0.05 was considered statistically significant. Additionally, odds ratio (OR) and corresponding 95 % confidence intervals (CIs) were calculated. Graphs were generated using GraphPad Prism 8 (GraphPad Software Inc., San Diego, CA, USA).

## 3 Results

### 3.1 Characteristics of the participants

A total of 7556 participants were enrolled in this study, of whom 59.18 % (4472/7556) were women and 40.82 % (3084/7556) were men. Among the ANA positive population, the proportion of females was 82.7 %, significantly higher than that of males at 17.3 %. The median and interquartile ranges (IQR) of ANA positive and negative ages were 38 (32–45) and 39 (32–46), respectively, with no significant difference (p = 0.424). In our study, participants who were ANA positive exhibited higher levels of TPOAb (14.55 IU/ml (IQR; 11.4–26.7) vs 12.4 IU/ml (IQR; 9.53–16.2), p < 0.001), TGAb (18.9 IU/ml (IQR; 17–88.93) vs 17.2 IU/ml (IQR; 15.7–69.28), p < 0.001) and TSH (2.18 mIU/ml (IQR; 1.48–3.27) vs 2.02 mIU/ml (IQR; 1.45–2.79), p = 0.002) compared to the ANA negative group. ANA positive group had a lower levels of fT3 (4.52 pmol/ml (IQR; 4.18–4.92) vs 4.69 pmol/ml (IQR; 4.32–5.14), p < 0.001), and fT4 (16.4 pmol/ml (IQR; 15.2–18.02) vs 17.1 pmol/ml (IQR; 15.7–18.5), p < 0.001) compared to the ANA negative group (Table 1).

**Table 1:** Characteristics of participants according to different status of ANA.

Variables	ANA -	ANA +	P value
Number, n	7030	526	
Sex			
Male	2993 (42.6 %)	91 (17.3 %)	<0.001^a^
Female	4037 (57.4 %)	435 (82.7 %)	
Age, Median (IQR), years	38 (32–45)	39 (32–46)	0.424^b^
TPOAb, Median (IQR), IU/ml	12.4 (9.53–16.2)	14.55 (11.4–26.7)	<0.001^b^
TgAb, Median (IQR), IU/ml	17.2 (15.7–69.28)	18.9 (17–88.93)	<0.001^b^
ft3, Median (IQR), png/ml	4.69 (4.32–5.14)	4.52 (4.18–4.92)	<0.001^b^
ft4, Median (IQR), png/ml	17.1 (15.7–18.5)	16.4 (15.2–18.02)	<0.001^b^
TSH, Median (IQR), μIU/ml	2.02 (1.45–2.79)	2.18 (1.48–3.27)	0.002^b^

ANA: antinuclear antibody; TPOAb: anti-thyroid peroxidase antibody; TgAb: anti-thyroglobulin antibody; fT3: free triiodothyronine; fT4: free thyroxine; TSH, thyroid-stimulating hormone. ^a^Chi-square test; ^b^Mann-Whitney *U* test was adopted.

### 3.2 Association between ANA status and thyroid autoantibodies

**Fig. 2:**
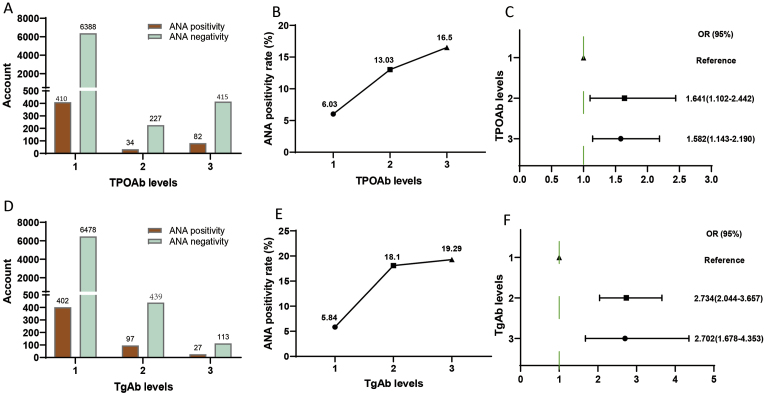
ANA and TPOAb/TgAb levels. (A, D) Comparison of the ANA positivity and negativity rates at different TPOAb/TgAb levels; orange and cyan represent ANA positivity and negativity, respectively. (B, E) ANA positivity rate at different TPOAb/TgAb levels. (C, F) Correlation between TPOAb/TgAb levels and ANA positivity risk. ANA, anti-nuclear antibody; TPOAb, thyroid peroxidase antibodies; TgAb, thyroglobulin antibody; CI: confidence interval; OR, odds ratio.

There were 410 (6.03 %), 34 (13.03 %) and 82 (16.5 %) participants with TPOAb levels 1, 2, and 3, respectively, who were positive for ANA. The positive proportion shows an upward trend (Fig. 2A and B). The ANA positivity rate varied significantly among the different TPOAb levels (χ^2^ = 93.711, P = 4.08 × 10^−21^), and there was a correlation between TPOAb levels and the ANA positivity rate (Kendall's tau-b = −0.109, P = 1.09 × 10^−11^) (Table 2). Regression analysis demonstrated a significant positive correlation between TPOAb levels and the risk of ANA positivity. The ANA positivity risk at TPOAb level 2 was 64.1 % higher than that at TPOAb level 1 (OR = 1.641; 95 % CI = 1.102–2.442). Similarly, the ANA positivity risk at TPOAb level 3 was 58.2 % higher than that at TPOAb level 1 (OR = 1.582; 95 % CI = 1.143–2.190) (Fig. 2C).

**Table 2:** Comparison of the ANA positivity rate at different TPOAb and TgAb levels.

TPOAb (IU/ml)	ANA positivity	ANA negativity	Chi-square test	Kendall's tau-b
Level 1 (≤34) n = 6798	410 (6.03 %)	6388 (93.97 %)	χ^2^ = 93.711 P = 4.08 × 10^−21^	Kendall's tau-b = −0.109 P = 1.09 × 10^−11^
Level 2 (34–100) n = 261	34 (13.03 %)	227 (86.98 %)		
Level 3 (≥100) n = 497	82 (16.5 %)	415 (83.5 %)		
Level 1 vs Level 2	χ^2^ = 20.869, OR = 0.429, 95 %Cl = 0.295–0.623, p = 0.000005			
Level 1 vs Level 3	χ^2^ = 80.688, OR = 0.325, 95 %Cl = 0.251–0.420, p = 2.6433 × 10^−19^			
Level 2 vs Level 3	χ^2^ = 1.592, OR = 0.758, 95 %Cl = 0.492–1.167, p = 0.207			

**TgAb (IU/ml)**	**ANA positivity**	**ANA negativity**	**Chi-square test**	**Kendall's tau-b**

Level 1 (≤115) n = 6880	402 (5.84 %)	6478 (94.16 %)	χ^2^ = 118.739 P = 5.03 × 10^−33^	Kendall's tau-b = −0.139 P = 1.76 × 10^−15^
Level 2 (115–500) n = 536	97 (18.10 %)	439 (81.90 %)		
Level 3 (≥500) n = 140	27 (19.29 %)	113 (80.71 %)		
Level 1 vs Level 2	χ^2^ = 118.976, OR = 0.281, 95 %Cl = 0.221–0.358, p = 1.06 × 10^−27^			
Level 1 vs Level 3	χ^2^ = 43.213, OR = 0.26, 95 %Cl = 0.169–0.4, p = 4.9086 × 10^−11^			
Level 2 vs Level 3	χ^2^ = 0.105, OR = 0.925, 95 %Cl = 0.576–1.485, p = 0.746			

ANA: antinuclear antibody; TPOAb: anti-thyroid peroxidase antibody; TgAb: anti-thyroglobulin antibody.

There were 402 (5.84 %), 97 (18.10 %) and 27 (19.29 %) participants with TgAb levels 1, 2, and 3, respectively, who were positive for ANA. The positive proportion shows an upward trend (Fig. 2D and E). The ANA positivity rate varied significantly among the different TgAb levels (χ^2^ = 118.739, P = 5.03 × 10^−33^), and there was a correlation between TgAb levels and the ANA positivity rate (Kendall's tau-b = −0.139, P = 1.76 × 10^−15^) (Table 2). Regression analysis demonstrated a significant positive correlation between TgAb levels and the risk of ANA positivity. The ANA positivity risk at TgAb level 2 was 173.4 % higher than that at TgAb level 1 (OR = 2.734; 95 % CI = 2.044–3.657). Similarly, the ANA positivity risk at TgAb level 3 was 170.02 % higher than that at TgAb level 1 (OR = 2.702; 95 % CI = 1.678–4.353) (Fig. 2F). Age-stratified analysis confirmed a stronger association between thyroid autoantibodies (TPOAb, TgAb) and ANA positivity in the younger cohort (<39 years). This was particularly evident at the highest antibody levels, where ANA positivity in the younger group reached 19.43 % for TPOAb and 24.66 % for TgAb, notably higher than the 13.6 % and 13.43 % observed in the older group (≥39 years) (Supp. Fig. S1).

### 3.3 Association between ANA status and thyroid hormones

**Fig. 3:**
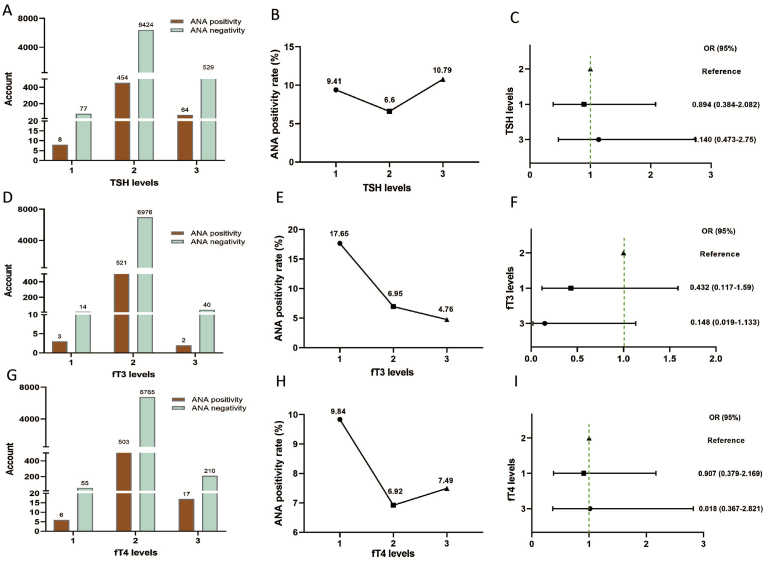
ANA and TSH/fT3/fT4 levels. (A, D, G) Comparison of the ANA positivity and negativity rates at different TSH/fT3/fT4 levels; orange and cyan represent ANA positivity and negativity, respectively. (B, E, H) ANA positivity rate at different TSH/fT3/fT4 levels. (C, F, I) Correlation between TSH/fT3/fT4 levels and ANA positivity risk. ANA, anti-nuclear antibody; TSH, thyroid-stimulating hormone; fT3, free triiodothyronine; fT4, free thyroxine; CI: confidence interval; OR, odds ratio.

There were 8 (9.41 %), 454 (6.6 %), and 64 (10.79 %) participants with positive ANA in TSH levels 1, 2, and 3, respectively (Fig. 3A and B). The ANA positivity rate varied significantly among different TSH levels (χ^2^ = 15.608, P = 4.08 × 10^−4^), However, Kendall's tau-b correlation showed a weak negative association between TSH levels and ANA positivity rate (tau-b = −0.037, P = 0.007) (Table 3). Logistic regression analysis indicated that high TSH levels had a marginal association with ANA positivity risk. The ANA positivity risk at TSH level 3 increased by 14 % compared with that at TSH level 2 (OR = 1.14, 95 % CI = 0.473–2.75) (Fig. 3C).

**Table 3:** Comparison of the ANA positivity rate at different TSH, fT3 and fT4 levels.

TSH (mIU/ml)	ANA positivity	ANA negativity	Chi-square test	Kendall's tau-b
Level 1 (≤0.27) n = 85	8 (9.41 %)	77 (90.59 %)	χ^2^ = 15.608 P = 0.000408	Kendall's tau-b = −0.037 P = 0.007
Level 2 (0.27–4.2) n = 6878	454 (6.60 %)	6424 (93.40 %)		
Level 3 (≥4.2) n = 593	64 (10.79 %)	529 (89.21 %)		
Level 1 vs Level 2	χ^2^ = 1.071, OR = 1.47, 95 %Cl = 0.705–3.064, p = 0.301			
Level 1 vs Level 3	χ^2^ = 0.149, OR = 0.859, 95 %Cl = 0.396–1.86, p = 0.699			
Level 2 vs Level 3	χ^2^ = 14.866, OR = 0.584, 95 %Cl = 0.443–0.770, p = 0.000115			

**fT3 (pmol/L)**	**ANA positivity**	**ANA negativity**	**Fisher's exact test**	**Kendall's tau-b**

Level 1 (≤3.1) n = 17	3 (17.65 %)	14 (82.35 %)	P = 0.214	Kendall's tau-b = 0.016 P = 0.201
Level 2 (3.1–6.8) n = 7497	521 (6.95 %)	6976 (93.05 %)		
Level 3 (≥6.8) n = 42	2 (4.76 %)	40 (95.24 %)		
**fT4 (pmol/L)**	**ANA positivity**	**ANA negativity**	**Fisher's exact test**	**Kendall's tau-b**

Level 1 (≤12) n = 61	6 (9.84 %)	55 (90.16 %)	P = 0.515	Kendall's tau-b = 0.001 P = 0.012
Level 2 (12–22) n = 7268	503 (6.92 %)	6765 (93.08 %)		
Level 3 (≥22) n = 227	17 (7.49 %)	210 (92.51 %)		

ANA: antinuclear antibody; fT3: free triiodothyronine; fT4: free thyroxine; TSH: thyroid-stimulating hormone.

There were 3 (17.65 %), 521 (6.95 %), and 2 (4.76 %) participants with positive ANA in fT3 levels 1, 2, and 3, respectively (Fig. 3D and E). The ANA positivity rate increased as fT3 levels decreased. There was no statistically significant difference in the ANA positivity rate among different fT3 levels (P = 0.214). Furthermore, there was no correlation between fT3 levels and ANA positivity rate (Kendall's tau-b = 0.016, P = 0.201) (Table 3; Fig. 3F).

There were 61 (9.84 %), 503 (6.92 %), and 17 (7.49 %) participants with positive ANA in fT4 levels 1, 2, and 3, respectively (Fig. 3G and H). Similar to the above-mentioned fT3 result. There was no statistically significant difference in the ANA positivity rate among different fT4 levels (P = 0.515). Furthermore, there was no correlation between fT4 levels and ANA positivity rate (Kendall's tau-b = 0.001, P = 0.012) (Table 3; Fig. 3I).

### 3.4 The ANA patterns and titers of the participants

Among 526 ANA-positive individuals, 494 nuclear staining patterns and 72 cytoplasmic staining patterns were detected (Table 4). The two most common nuclear staining patterns were the AC-4/5 (fine or large speckled) at 37.64 % and AC-1 (homogeneous) at 27.95 %. The most common cytoplasmic patterns was AC-19/20 (cytoplasmic speckled). Furthermore, the most frequent ANA titer was 1:100 (57.95 %), followed by 1:320 (27.74 %).

**Table 4:** Nuclear and cytoplasmic staining patterns and titers of antinuclear antibodies in participants (n = 526).

N (%)	participants
Nuclear staining patterns of ANA	

AC-1 (homogeneous)	147 (27.95 %)
AC-2 (dense fine speckled/dfs-70)	59 (11.22 %)
AC-3 (centromere)	21 (3.99 %)
AC-4/5 (fine or large speckled)	198 (37.64 %)
AC-6/7 (few or multiple nuclear dots)	16 (3.04 %)
AC-8/9/10 (homogeneous, clumpy, or punctate nucleolar)	44 (8.37 %)
AC-11/12 (punctate or smooth nuclear envelope)	9 (1.71 %)

Cytoplasmic staining patterns of ANA	

AC-15/16/17 (fibrillar linear, filamentous, or segmental)	9 (1.71 %)
AC-19/20 (speckled)	49 (9.32 %)
AC-21 (reticular/AMA)	10 (1.90 %)
AC-22 (polar/Golgi-like)	3 (0.57 %)
AC-23 (rods and rings)	1 (0.19 %)

Titers of ANA	

1:100	328 (57.95 %)
1:320	157 (27.74 %)
1:1000	79 (13.96 %)
1:3200	1 (0.18 %)
1:10000	1 (0.18 %)

ANA: antinuclear antibody; AC: anti-cell; AMA: anti-mitochondrial antibodies; dfs: dense fine speckled.

**Fig. 4:**
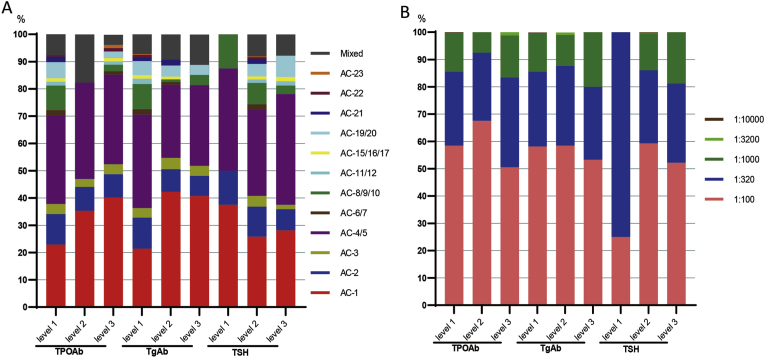
(A) The percentage of individual ANA patterns observed in different TPOAb/TgAb/TSH/fT3/fT4 levels. (B) The percentage of individual ANA titers determined by IIFA in different TPOAb/TgAb/TSH/fT3/fT4 levels. ANA, anti-nuclear antibody; TPOAb, thyroid peroxidase antibodies; TgAb, thyroglobulin antibody; TSH, thyroid-stimulating hormone; IIFA, the indirect immunofluorescence assay; AC: anti-cell.

We described the distribution of ANA patterns across different concentration levels of TPOAb, TgAb, and TSH (Table 5). The proportion of the AC-1 pattern increased with higher concentrations of both TPOAb and TgAb (Fig. 4A). The proportion of AC-1 was also relatively high in samples with abnormal TSH concentrations. The predominant ANA pattern for TPOAb level 1 was AC-4/5 (32.4 %). For TPOAb level 2, the predominant patterns were AC-1 (35.3 %) and AC-4/5 (35.3 %). For TPOAb level 3, the predominant pattern was AC-1 (40.2 %). At TgAb level 1, the predominant pattern was AC-4/5 (34.3 %), while at TgAb levels 2 and 3, the predominant pattern was AC-1 (42.3 % and 40.7 %, respectively). AC-4/5 was the predominant pattern across all three TSH levels. Mixed ANA patterns were observed at nearly all concentration levels of the three indicators, except for the low concentration level of TSH where none were detected. Consistent with the overall analysis, the most frequent ANA titer across all concentration levels of TPOAb, TgAb, and TSH was 1:100, followed by 1:320. The exception was the low TSH concentration level, where 1:320 was the most frequent titer (Table 6; Fig. 4B).

**Table 5:** ANA patterns at different levels of TPOAb, TgAb and TSH.

ANA pattern	TPOAb (IU/ml)			TgAb (IU/ml)			TSH (mIU/ml)		
	Level 1 (≤34)	Level 2 (34–100)	Level 3 (≥100)	Level 1 (≤115)	Level 2 (115–500)	Level 3 (≥500)	Level 1 (≤0.27)	Level 2 (0.27–4.2)	Level 3 (≥4.2)
AC-1	94 (22.9 %)	12 (35.3 %)	33 (40.2 %)	86 (21.4 %)	41 (42.3 %)	11 (40.7 %)	3 (37.5 %)	117 (25.8 %)	18 (28.1 %)
AC-2	46 (11.2 %)	3 (8.8 %)	7 (8.5 %)	46 (11.4 %)	8 (8.3 %)	2 (7.4 %)	1 (12.5 %)	50 (11 %)	5 (7.8 %)
AC-3	15 (3.7 %)	1 (2.9 %)	3 (3.7 %)	14 (3.5 %)	4 (4.1 %)	1 (3.7 %)	0	18 (4 %)	1 (1.6 %)
AC-4/5	133 (32.4 %)	12 (35.3 %)	27 (32.9 %)	138 (34.3 %)	26 (26.8 %)	8 (29.6 %)	3 (37.5 %)	143 (31.5 %)	26 (40.6 %)
AC-6/7	8 (2.0 %)	0	1 (1.2 %)	8 (2.0 %)	1 (1 %)	0	0	9 (2 %)	0
AC-8/9/10	37 (9.0 %)	0	2 (2.4 %)	37 (9.2 %)	1 (1 %)	1 (3.7 %)	1 (12.5 %)	36 (7.9 %)	2 (3.1 %)
AC-11/12	6 (1.5 %)	0	1 (1.2 %)	8 (2.0 %)	0	0	0	6 (1.3 %)	1 (1.6 %)
AC-15/16/17	5 (1.2 %)	0	1 (1.2 %)	5 (1.2 %)	1 (1 %)	0	0	5 (1.1 %)	1 (1.6 %)
AC-19/20	24 (5.9 %)	0	2 (2.4 %)	21 (5.2 %)	4 (4.1 %)	1 (3.7 %)	0	21 (4.6 %)	5 (7.8 %)
AC-21	8 (2.0 %)	0	0	6 (1.5 %)	2 (2.1 %)	0	0	8 (1.8 %)	0
AC-22	2 (0.5 %)	0	1 (1.2 %)	3 (0.8 %)	0	0	0	3 (0.7 %)	0
AC-23	0	0	1 (1.2 %)	1 (0.3 %)	0	0	0	1 (0.2 %)	0
mix	32 (7.8 %)	6 (17.7 %)	3 (3.7 %)	29 (7.2 %)	9 (9.3 %)	3 (11.1 %)	0	37 (8.1 %)	5 (7.8 %)

ANA: antinuclear antibody; TPOAb: anti-thyroid peroxidase antibody; TgAb: anti-thyroglobulin antibody; TSH: thyroid-stimulating hormone; AC: anti-cell. *Note*: Data were expressed as number (percentage).

**Table 6:** ANA titers at different levels of TPOAb, TgAb and TSH.

ANA titers	TPOAb (IU/ml)			TgAb (IU/ml)			TSH (mIU/ml)		
	Level 1 (≤34)	Level 2 (34–100)	Level 3 (≥100)	Level 1 (≤115)	Level 2 (115–500)	Level 3 (≥500)	Level 1 (≤0.27)	Level 2 (0.27–4.2)	Level 3 (≥4.2)
1:100	258 (58.5 %)	27 (67.5 %)	43 (50.6 %)	250 (58.1 %)	62 (58.5 %)	16 (53.3 %)	2 (25 %)	290 (59.3 %)	36 (52.2 %)
1:320	119 (27 %)	10 (25 %)	28 (32.9 %)	118 (27.4 %)	31 (29.2 %)	8 (26.7 %)	6 (75 %)	131 (26.8 %)	20 (29 %)
1:1000	63 (14.3 %)	3 (7.5 %)	13 (15.3 %)	61 (14.2 %)	12 (11.3 %)	6 (20 %)	0	66 (13.5 %)	13 (18.8 %)
1:3200	0	0	1 (1.2 %)	0	1 (0.9 %)	0	0	1 (0.2 %)	0
1:10000	1 (0.2 %)	0	0	1 (0.2 %)	0	0	0	1 (0.2 %)	0

ANA: antinuclear antibody; TPOAb: anti-thyroid peroxidase antibody; TgAb: anti-thyroglobulin antibody; TSH: thyroid-stimulating hormone. *Note*: Data were expressed as number (percentage).

## 4 Discussion

This large-scale retrospective study, encompassing 7556 individuals, provides compelling evidence for a significant association between elevated thyroid autoantibody levels and an increased risk of ANA positivity, while revealing more complex and generally weaker relationships with thyroid hormone levels. Our findings reinforce the interconnectedness of autoimmune processes targeting different organ systems.

ANAs are a series of autoantibodies that can react with various nuclear molecules, including deoxyribonucleic acid (DNA), ribonucleic acid, histones, acidic nucleoproteins, or complexes of these molecular elements [15]. In our study, the positive rate of ANA in the physical examination population was 7 %, similar to a previous study **tha**t reported an ANA positivity rate of 9 % in healthy controls [16]. Previous studies have confirmed that the positive rates of TPOAb and ANA in the elderly population of the community are both relatively high [17]. Additionally, there are reports in the literature that anti-dsDNA is positively correlated with TPOAb [18]. Some studies have also concluded that positive ANA is related to HT [19]. However, up to now, there have been few analyses of the relationship between the positive rate, pattern and titer of ANA and the clinical indicators of HT and GD in the healthy physical examination population.

The striking female predominance (82.7 %) within the ANA-positive cohort strongly echoes the well-established female predisposition to autoimmune diseases in general, including both AITDs and SARDs [20]. This reinforces the critical role of sex hormones and potentially X-chromosome-related factors in immune tolerance [21, 22]. The lack of significant age difference between groups suggests that within this adult cohort, age was not a major confounder for the specific associations studied.

Our investigation demonstrated increasing ANA positivity rates with higher TPOAb levels: 6.03 % (TPOAb ≤34 IU/ml), 13.03 % (34 IU/ml < TPOAb <100 IU/ml), and 16.50 % (TPOAb ≥100 IU/ml). A similar trend was observed for TgAb: 5.84 % (TgAb ≤115 IU/ml), 18.10 % (115 IU/ml < TgAb <500 IU/ml), and 19.29 % (TgAb ≥500 IU/ml). Notably, both the positivity rate and risk of ANA were higher at elevated TgAb levels compared to elevated TPOAb levels. This suggests distinct pathophysiological mechanisms underlie these thyroid autoantibodies despite their shared association with AITD [23]. Participants with higher TPOAb levels exhibited significantly increased odds of ANA positivity (OR = 1.64 and 1.58, respectively) compared to those with the lowest levels. This trend was even more pronounced for TgAb, where Levels 2 and 3 conferred substantially higher risks (OR = 2.73 and 2.70, respectively). The highly significant chi-square results (P < 10^−21^ for TPOAb, P < 10^−33^ for TgAb) and significant negative Kendall's tau-b correlations (indicating increasing ANA positivity with increasing autoantibody category) further solidify this relationship. The results of our age-stratified analysis confirm that the link between thyroid autoimmunity and systemic ANA positivity is a robust finding, observable across different adult age groups. The stronger association observed in the younger cohort (<39 years) is a notable finding. This may reflect a more active or susceptible immune system in younger individuals. Given TPOAb and TgAb serve as biomarkers for HT, our data further support an association between ANA positivity and HT pathogenesis. These results strongly suggest that the presence and magnitude of thyroid-specific autoimmunity, as reflected by TPOAb and particularly TgAb titers, are associated with a heightened propensity for systemic autoimmunity marked by ANA.

In contrast, the associations between thyroid hormone levels and ANA status were notably weaker. While TSH levels showed a statistically significant difference in ANA positivity rates across categories (P = 0.000408) and a weak negative Kendall's tau-b correlation, the clinical relevance of the marginal increase in ANA positivity risk at TSH ≥4.2 mIU/L (OR = 1.14, 95 % CI = 0.473–2.75) is questionable, especially given the wide confidence interval crossing 1. However, some studies suggest that ANA positivity is more likely to occur in patients with elevated TSH levels, such as patients with hypothyroidism [7, 24]. Similarly, although fT3 levels were lower in the ANA-positive group overall, the analysis across categorized fT3 and fT4 levels revealed no statistically significant differences in ANA positivity rates (P = 0.214 and P = 0.515, respectively) and no significant correlations. This dissociation implies that while thyroid autoimmunity (autoantibodies) is linked to systemic autoimmunity, the resultant thyroid dysfunction (hormone abnormalities) may not be the primary driver of ANA positivity in this population.

The predominant ANA staining pattern in our positive cohort was nuclear. The most frequent specific patterns were AC-4/5 (fine or large speckled; 37.64 %) and AC-1 (homogeneous; 27.95 %). The majority of ANA-positive individuals exhibited low-titer immunofluorescence, consistent with characteristics of a general examination population. The shift in the predominant ANA pattern from AC-4/5 (fine/large speckled) in the lowest TPOAb and TgAb categories to AC-1 (homogeneous) in the higher categories is intriguing. The AC-1 pattern, often associated with anti-dsDNA and anti-chromatin antibodies in conditions like SLE [25, 26], was increasingly prevalent as thyroid autoantibody concentrations rose. This pattern shift, particularly evident at the highest TPOAb and TgAb levels (40.2 % and 40.7 % AC-1, respectively), may reflect an evolution in the autoimmune response or indicate a subgroup of patients with a more systemic autoimmune phenotype co-existing with significant thyroid autoimmunity. The predominance of the AC-4/5 pattern across all TSH levels suggests that thyroid dysfunction per se may not strongly influence the specific ANA pattern. The consistency of the most frequent titers (1:100 and 1:320) across most marker levels further emphasizes the link between autoantibody burden and ANA pattern rather than titer in this context. These specific pattern associations reinforce the links between HT and ANA pattern. Diagnosing HT merely by interpreting the positive pattern of ANA is not rigorous. We still need more clinical evidence to support the relevant conclusion, but at least it can enable the laboratory personnel to observe the possibility of specific ANA patterns later suggesting thyroid-related diseases.

## 5 Limitations

Due to its retrospective design, which is the significant limitation of our study. We lack detailed clinical diagnoses for the participants, preventing us from correlating these serological findings with specific autoimmune diseases (e.g., Hashimoto's thyroiditis, Graves' disease, SLE, Sjögren's syndrome). The study population is from a single center, and the generalizability of findings to other ethnicities or healthcare settings requires further investigation. Data on potential confounders like medication use (e.g., thyroid hormone replacement, immunosuppressants) or other autoimmune markers were not available. In this study's screening cohort, specific autoantibody testing was not performed; reliance solely on ANA screening by indirect immunofluorescence (IFA) may yield insufficient specificity and potential misdiagnosis. Therefore, individuals in this cohort screening positive for high-titer ANA should be referred to rheumatology or hematology for comprehensive specific autoantibody testing to confirm diagnosis.

## 6 Conclusions

This study robustly demonstrates that elevated levels of thyroid autoantibodies, TPOAb and especially TgAb, are significantly associated with an increased risk of ANA positivity in a large clinical cohort. The association with thyroid hormone levels was substantially weaker. The observed shift towards the AC-1 (homogeneous) ANA pattern at higher thyroid autoantibody concentrations may signify a distinct serological profile warranting further investigation. Our findings suggest that patients presenting with high titers of TPOAb or TgAb, particularly women, represent a population where ANA testing and vigilance for potential systemic autoimmune features may be warranted. Future prospective studies linking these serological markers to specific clinical diagnoses are needed to fully elucidate the clinical implications of this association.

## Funding

This work was supported in part by grants from the 10.13039/501100001809National Natural Science Foundation of China (No. 82202498), and Qingpu Branch of the 10.13039/501100010108Zhongshan Hospital (QYM2023-04).

## CRediT authorship contribution statement

**Mengjiao Yuan:** Funding acquisition, Investigation, Methodology, Resources, Software, Writing – original draft. **Jinjin Wang:** Data curation, Formal analysis, Software, Supervision, Writing – original draft. **Wanjun Yu:** Data curation, Formal analysis, Software. **Jianmin Lin:** Data curation, Investigation, Resources. **Lei Yue:** Conceptualization, Data curation, Formal analysis, Funding acquisition, Methodology, Resources, Supervision, Visualization, Writing – review & editing. **Qian Gong:** Methodology, Resources, Supervision, Writing – review & editing.

## Declaration of competing interest

The authors declare that they have no known competing financial interests or personal relationships that could have appeared to influence the work reported in this paper.

## Figures and Tables

**figs1:**
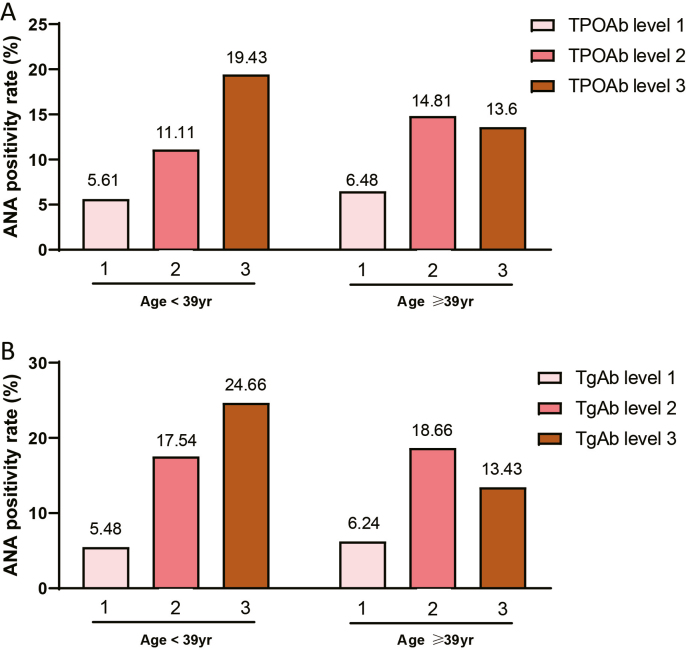


## Data Availability

Data are available from the corresponding author upon reasonable request.
